# The role of machine learning in health policies during the COVID-19 pandemic and in long COVID management

**DOI:** 10.3389/fpubh.2023.1140353

**Published:** 2023-04-11

**Authors:** Lindybeth Sarmiento Varón, Jorge González-Puelma, David Medina-Ortiz, Jacqueline Aldridge, Diego Alvarez-Saravia, Roberto Uribe-Paredes, Marcelo A. Navarrete

**Affiliations:** ^1^Centro Asistencial Docente y de Investigación, Universidad de Magallanes, Punta Arenas, Chile; ^2^Escuela de Medicina, Universidad de Magallanes, Punta Arenas, Chile; ^3^Departamento de Ingeniería en Computación, Facultad de Ingeniería, Universidad de Magallanes, Punta Arenas, Chile

**Keywords:** COVID-19, public health policies, mathematical models, machine learning, long COVID, SARS-CoV-2

## Abstract

The ongoing COVID-19 pandemic is arguably one of the most challenging health crises in modern times. The development of effective strategies to control the spread of SARS-CoV-2 were major goals for governments and policy makers. Mathematical modeling and machine learning emerged as potent tools to guide and optimize the different control measures. This review briefly summarizes the SARS-CoV-2 pandemic evolution during the first 3 years. It details the main public health challenges focusing on the contribution of mathematical modeling to design and guide government action plans and spread mitigation interventions of SARS-CoV-2. Next describes the application of machine learning methods in a series of study cases, including COVID-19 clinical diagnosis, the analysis of epidemiological variables, and drug discovery by protein engineering techniques. Lastly, it explores the use of machine learning tools for investigating long COVID, by identifying patterns and relationships of symptoms, predicting risk indicators, and enabling early evaluation of COVID-19 sequelae.

## 1. Introduction

Mathematical models help to understand the functioning and dynamics of a given system trough equations and rules, as such, can simulate conditions and scenarios associated with multiple public policies, non-pharmaceutical interventions (NPI), and vaccine performance (1). Therefore, mathematical models became major tools for guiding the decision-making of governments and health systems during the pandemic (2). This section briefly introduces SARS-CoV-2 (Severe Acute Respiratory Syndrome Coronavirus 2) and describes relevant events during the progress pandemic. We then summarize the main applications of mathematical models and the various uses to describe the transmission behaviors of SARS-CoV-2.

### 1.1. What is the SARS-CoV-2?

SARS-CoV-2 is the pathogen causing the 2019 coronavirus disease (COVID-19). COVID-19 manifestations range from mild flu symptoms to severe acute respiratory syndrome. SARS-CoV-2 virion contains a 29Kb RNA genome wrapped in a capsid covered by the Spike, the main protein responsible for the high infection rate (3). During the transmission between humans, the genome accumulates mutations, generating variants with selective advantages that predominate in different countries (4). Intrinsic factors like transmissibility and natural mutation rate, host factors such as age, risk group, immunity, and socio-cultural factors like economy, culture, and current levels of globalization have determined the coronavirus evolution. Integrating SARS-CoV-2 data is essential to predict its behavior, prevent its continuous expansion, and understand this disease. Three years after the pandemic, the scientific community has generated an unprecedented amount of data, now facing the challenge of translating this data into knowledge.

### 1.2. The first 3 years of the pandemic

The first reported case of COVID-19 was in December 2019 (5). With the exponential increase in infections worldwide, the World Health Organization (WHO) declared the disease a pandemic in March 2020. Governments adopted different NPI to mitigate the virus's high reproduction rates. These measures included face masks, social distancing, and lockdowns. While these measures were implemented worldwide, just a few countries, such as Vietnam and New Zealand, demonstrated the complete -although transitory- elimination of the transmission (6). In April and May 2020, the first predictions of the pandemic course were based on statistical models performed by the Institute for Health Metrics and Evaluation and provided a reasonable projection in the short-time (7). During the first wave, it was also possible to establish that 10% of the cases were responsible for 80% of the secondary infections, indicating a high heterogeneity in transmission spread as compared to other pathogens (8).

In the first pandemic year, it was identified that social contact in public transport or closed areas allowed high transmission rates (9, 10). In turn, it was determined that face masks reduce droplet particle transmission (11). Furthermore, NPI was essential to flatten the spread curve in the first year of the pandemic preventing new waves of cases after curves pick, limiting overcrowding of hospital beds, and giving time to improve treatment strategies (12). Adaptations of the Susceptible-Infected-Recovered models helped to demonstrate the NPI effectiveness in preventing the transmission of the virus. Besides, these same models allowed the detection of an increase in virus circulation with the relaxation of the measures (13). Other models, facilitated the test-track-isolation developing strategies to prevent the spread, demonstrating that efficient track strategies help to reduce the number of new cases (14). At the same time, the first signs of SARS-CoV-2 genetic adaptation arose between March and May 2020, with the emerging D614G variant, which showed clear worldwide transmissibility advantages (15). The control of the pandemic at that time relied on the development of herd immunity, being established that the necessary protection of the population is approximately $1-\frac{1}{R_{0}}$, being estimated at 67% of the people (16). In August 2020, reinfection cases demonstrated that natural immunity only provides temporary protection (17). In December 2020, the first clinical trials of vaccines were developed, leading to the emergency approval of traditional and novel vaccine formulations -such as mRNA vaccines-. These studies quickly established that immunity begins between 10 and 14 days after the first dose (18). A second dose shows protection over 90%, preventing hospitalizations and deaths (19). The vaccines can block propagation, making cases less infectious, with a 92% reduction in transmission rates (20). At the same time, quantitative models pointed to the possibility of immune escape when complete schemes are not generated. At the end of 2020, the Alpha variant (B.1.1.7), according to the WHO terminology, was the variant responsible for the significant increase in cases in the United Kingdom. This variant was characterized by presenting spike mutations with binding advantages to the ACE-2 receptor (21), showing clear selection advantages, a phenomenon observed simultaneously in different parts of the world (22, 23).

**Figure 1:**
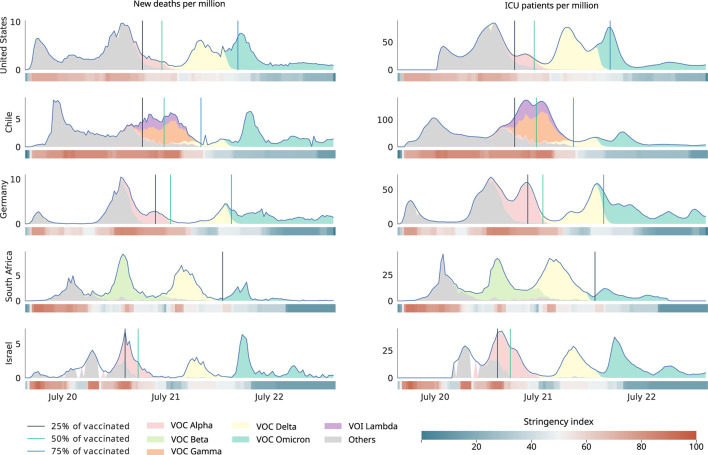
Behavior of epidemiological variables during the ongoing pandemic of COVID-19. The figure depicts the timeline of new deaths per million inhabitants **(left)** and admissions to intensive care unit (ICU) per million inhabitants **(right)** in relationship with SARS-CoV-2 variants, vaccination thresholds, and non-pharmacological interventions. The stringency index is a composite measure based on nine response indicators including school closures, workplace closures, and travel bans, rescaled to a value from 0 to 100 (100 = strictest). Data acquired from et al. (27), Hasell et al. (28), and Khare et al. (29).

The subsequent variant of similar global relevance was Delta (B.1.617.2), characterized by its high replicative capacity. Vaccine effectiveness studies showed protection against Alpha and Delta variants (24). Vaccination programs were effective reducing deaths, hospitalization admission, and intensive care unit (ICU) occupancy (see Figure 1). In November 2021, a new outbreak was reported in South Africa, caused by a new circulating variant presenting a 60–70 spike gene deletion. This variant was called Omicron (B1.1.529) and expanded rapidly throughout the world, replacing the Delta variant. Omicron carries more than 30 spike mutations (25), being responsible for high worldwide reinfection rates (26). Vaccines have also shown a protective effect against this variant, although deaths were reported among unvaccinated individuals. Omicron subvariant (XBB1.5) has been described as responsible for 40.5% confirmed cases in the EE.UU. as of late December 2022. It has also been observed that recombinant XBB and BA.2 Omicron subvariant strains, widely spread in Asia, do not show different symptoms than the previous variants, nor do they show signs of being more severe than their predecessors.

Figure 1 summarizes the key variables depicting the pandemic evolution in five exemplary cases. Each country showed different spread behaviors of SARS-CoV-2. The measures showed variable effectiveness. In most countries, other public health policies and government plans were applied to mitigate the effects of the spread. However, in most cases, the fatalities decreased after implementation.

### 1.3. Applications of mathematical modeling during the pandemic

The SIR models (Susceptible, Infected, and Recovered) are spread dynamics analysis models used during the early days of the pandemic (30). SEIR models (Susceptible-Exposed-Infected-Recovered) correspond to an adapted SIR model to understand propagation mechanisms (31). These models do not account for heterogeneity within the population, thus novel strategies incorporated a component of population subdivision into multiple groups and interconnected systems, allowing the representation of several mechanisms of interaction between different sub-populations by a multi-group SEIRA (Susceptible-Exposed-Infected-Recovered and Asymptomatic Model) (32). Another interesting development was the statistically-based temporal reclassification of cases. This approach allowed more precise modeling of SARS-CoV-2 propagation dynamics, by correcting errors in diagnostic test reporting times and infection time registries (33, 34).

With the application of NPI strategies to prevent the spread of SARS-CoV-2, the mathematical models were adapted to incorporate this new knowledge. This adaptations enabled the anticipation of the effect of NPI relaxation measures in function of epidemiological variables, such as levels of hospitalization, use of ICU, and lethality (35). SEIRA models also helped to asses the effect of vaccines and pharmaceutical interventions (36).

With the first vaccination plans and high immunization rates started the relaxation of public policies (37). However, the ability of the virus to mutate and generate variants was associated with new peaks in cases incidence. Mathematical models were adapted to this scenario by incorporating information on genomic surveillance programs, spread of variants, and the effects of immunization (38–40).

Altogether, mathematical tools proved its relevance in modeling the behavior of propagation systems and their effect on populations. The SIR classical model as well as different adaptations such as SEIR, SEIRA, and others, contributed significantly to the development of government plans and public health policies. Nevertheless, traditional mathematical modeling strategies rely on existing knowledge and cannot account for dynamics not explicitly incorporated during modeling. Methods based on machine learning (ML) and artificial intelligence (AI) can overcome these intrinsic limitations by generating autonomous systems that learn from the modeled dynamics to predict new behaviors and adapt to unknown scenarios.

### 1.4. Vaccines developments, efficacy, and adverse effects

Population immunity is considered a landmark for epidemic control. Since immunity through natural infection might result in unacceptable morbidity and mortality, the development of efficient COVID-19 vaccination programs was a prioritary public policy for most countries (41, 42). The race to develop highly effective and safe vaccines resulted in various platforms allowing their implementation at unprecedented speed (43–45).

Due the modest response of traditional vaccines against other coronaviruses such as Middle East Respiratory Syndrome Coronavirus (MERS) and Severe Acute Respiratory Syndrome (SARS), the development of novel formulations was a major scientific goal (42, 46). A new vaccine technology based on mRNA technology emerged as candidates in late December 2020 and two formulations granted emergency approval BNT162b2 (Pfizer-BioNTech), and mRNA-1273 (Moderna) (47). The developed vaccines showed promising results in reducing transmissibility and the probability of death, reaching an efficacy > 90% in phase III clinical trials (48).

The widespread immunization poses the challenge of quantifying and understanding short- and long-term toxicity for novel vaccine formulations. Most studies have shown short-term safety in the general population. However, in certain groups, severe adverse events were reported i) anaphylaxis (2.5–4.8 cases per million adult vaccine doses administered) (49, 50), ii) myocarditis (52.4 cases and 56.3 cases per million doses) (51), iii) thrombosis with thrombocytopenia syndrome (2-4 cases per one million doses administered) (52), and iv) Guillain-Barré syndrome (7.8 cases per million) (53), as well as an association with multisystemic inflammatory syndrome (54).

A major challenge is to reliably detect long-term effects that might occur at different rates in different patients subgroups (55). Causal association becomes difficult due to the high immunization rates achieved in most countries. In this complex scenario mathematical models, ML, and AI, could provide powerful tools provided that public policies focus on collection of sufficient high-quality data.

### 1.5. What is long COVID?

#### 1.5.1. Characteristics and definitions of long COVID

Long COVID (LC) is a novel multi-systemic disease defined by the persistence or appearance of a wide variety of symptoms with variable intensity, regardless of the initial disease severity by probable or confirmed SARS-CoV-2 infection (56). In response to the absence of a consensus definition, the WHO proposed using the term Post-COVID-19 listed in the ICD-10 classification based on the Delphi consensus (57). This condition usually manifests 3 months after the SARS-CoV-2 infection, the symptoms last for at least 2 months in the absence of alternative diagnosis (58).

The National Institute for Health Research, classifies LC into i) post-intensive care syndrome (post-ICU syndrome), ii) post-viral fatigue syndrome, iii) permanent organ damage, iv) decompensation of previous chronic diseases, v) the onset of a new disease triggered by COVID-19, and vi) pharmacological toxicity from COVID-19 treatment (59).

Other authors had suggested six post-COVID syndrome subsets, including i) non-severe COVID-19 multiorgan sequelae, ii) pulmonary fibrosis sequelae, iii) myalgic encephalomyelitis/chronic fatigue syndrome, iv) postural orthostatic tachycardia syndrome, v) post-intensive care syndrome, and vi) medical or clinical sequelae (60).

#### 1.5.2. Symptoms and incidence of long COVID

**Figure 2:**
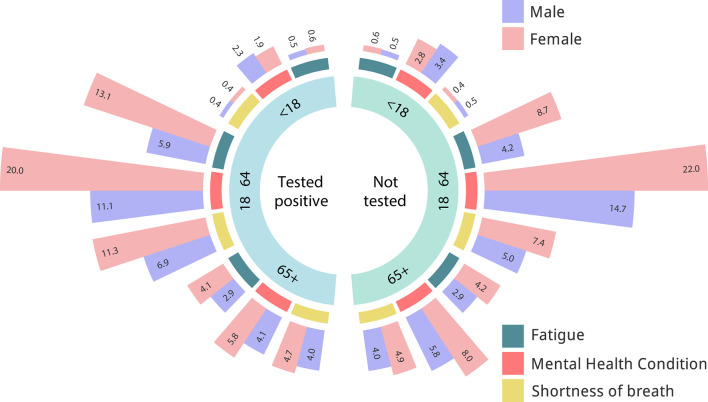
Analysis of long COVID symptoms in patients with positive or negative COVID-19 PCR test. Relative frequency of symptoms in individuals with a positive COVID-19 PCR test (left) as compared to individuals with a negative test. Elaborated on basis of LC symptoms registered by the National COVID Cohort Collaborative (N3C) initiative. Data acquired from (71).

Between 2.3 and 60% of COVID-19 survivors could experience LC symptoms during the first year, and up to 42% 2 years after the infection (61–63). Patients with LC present variable symptoms, including fatigue (29%), muscle pain, palpitations, cognitive impairment (28%), dyspnea (21%), anxiety (27%), chest pain, and arthralgia (18%) (see Figure 2) (64). Other patients report respiratory system dysfunction (26%), or cardiovascular complications (32–89%) 3 months after the onset of infection (65–67). Gastrointestinal symptoms have been associated with an imbalance of gut microbiota, as well as psychological and central nervous system effects (68, 69). Most of these symptoms are associated with a reduction in the quality of life. However, the distinction between SARS-CoV-2-related symptoms to those linked to other, often pre-existing conditions remains extremely challenging. As clinical studies addressing this issues take a long time to develop the NIH launched the Rapid Acceleration of Diagnostics initiative, and the NIH LC Computational Challenge (70). This initiative aims to use AI and ML to predict which patients with SARS-CoV-2 infections are most likely to develop LC. Figure 2 depicts the relative frequency of LC symptoms registered by the National COVID Cohort Collaborative (N3C) initiative. Inviduals that tested positive for SARS-CovV-2 show a higher frequency of alterations in symptoms such as fatigue and shortness of breath. The prevalence of these symptoms seems higher in women. However, the small magnitude of the differences highlights the challenge of differentiation long COVID from other conditions.

## 2. Machine learning application to COVID-19

**Figure 3:**
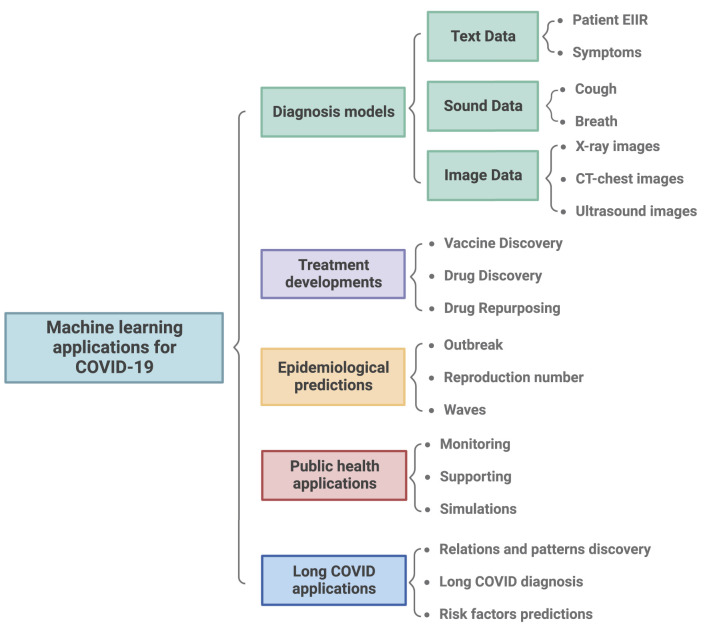
Summary of machine learning applications to fight COVID-19 during the pandemic. General applications of machine learning were classified into 5 categories: i) The design of diagnosis models based on different types of inputs like CT chest, X-ray images, and symptom descriptions. ii) Treatment development. iii) The development of epidemiological models to predict new waves and outbreaks. iv) The simulation of potential scenarios, and monitoring systems to guide public health decisions. v) The diagnosis and identification of risk factors in long COVID.

During the COVID-19 pandemic, ML methods have played a relevant role in the development of diagnostic strategies (72, 73), forecasting the epidemiological behavior (74), and as a tool to support the development and monitoring of public health policies (75). Figure 3 summarizes the most relevant ML applications during the COVID-19 pandemic.

### 2.1. COVID-19 diagnosis

Different strategies based on ML algorithms were designed during the COVID-19 pandemic to elaborate predictive models of efficient clinical diagnosis (76). The main inputs used to build the models are based on images, sounds, respiratory information, symptoms, and mixed data (77). Convolutional neural networks (CNN) architectures are commonly employed to develop classification models *via* image inputs (e.g., x-ray, CT-chest, and ultrasounds) (78). Sounds from respiratory information, such as cough and breath, were common inputs for the development of predictive models employing recurrent neural network (RNN) or long short-term memory architectures (LSTM), since this type of architectures have the advantage to maintain the information on signal frequencies (79). Hybrid methods that combine symptoms and clinical diagnostic tests as inputs facilitate the development of more complex predictions models or classifications systems. The hybrid methods include not only vector information or matrix spaces, but also data on disease's propagation. The incorporation of virus characteristics, close contacts, and contagion networks using graph neural networks results in highly efficient prediction systems (80).

**Figure 4:**
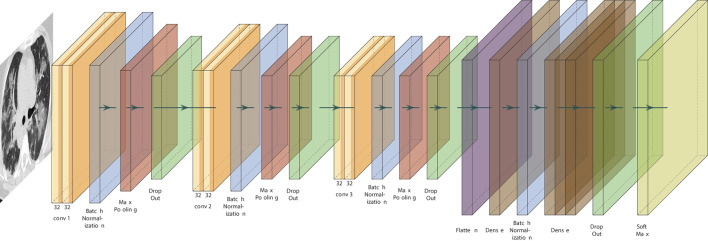
Developed architecture for COVID-19 diagnosis classification models based on CT chest images and convolutional neural network architectures. Three blocks of layers composed of convolution, batch normalization, max pooling and dropout layers are generated as a pattern extraction strategy, then a flatten layer is used to generate the inputs to the dense layers, which are joined with a layer of batch normalization, followed by three additional full connected layers, which end with a new dropout layer to prevent overfitting, and the final classification layer. ReLU is used as activation functions and the SoftMax function in the classification layer. Finally, the Adam optimizer is used as a loss function binary cross entropy. The developed architecture is an update from previous method for CT chest images classification models developed by our group (34).

To demonstrate the usability of classification models based on ML techniques, a clinical diagnostic model using CT chest images was developed following the architecture proposed in Figure 4 and updating our previously reported method for CT chest images classification (34). Generally, models based on CNN architectures can be divided into three large blocks: i) pattern processing and extraction, ii) learning, and iii) classification blocks. To extract patterns, a set of three layers composed of CNN, batch normalization, max pooling, and dropout, was developed. Then, a flattened layer is used to prepare the inputs to the fully connected or dense layers, which are part of the learning block, composed of dense layers interspersed with batch normalization, ending with a dropout layer. Finally, a last layer of classification is added to develop the outputs. As activation functions, ReLU and SoftMax were used. In addition, binary cross entropy associated with an Adam optimizer was used as a cost function. A total of 2,482 images were used to train the diagnostic model extracted from (81). For the training process, a classic validation approach was followed by segmentation of the training and validation data set (80:20), and the TensorFlow framework was employed for its implementation (82). Model training was followed for a total of 10 epochs. The proposed architecture achieved a precision of 99.81% and 0.027 loss function, demonstrating the high performance obtained by the proposed architecture. The implemented model can be used as a support strategy for clinical diagnosis in patients with COVID-19. Besides, it is possible to apply transfer learning techniques to use the same images and the same architecture proposed to estimate the probability that patients present sequelae, one of the most recent areas of study associated with the concept of LC.

### 2.2. COVID-19 treatments and strategies to prevent adverse effects

ML applications related to the design of treatment strategies have focused on drug discovery, drug repurposing, and vaccine discovery methods (83). For drug repurposing, algorithms are usually based on networks of knowledge graphs including virus and host interactions (84). These strategies have used particular network label propagation combined with semi-supervised learning method based on regularized Laplacian to identify interactors of SARS-CoV-2 (85). Another example is the elaboration of predictive systems based on protein-protein interaction to estimate affinity between two elements (86). This issue has been addressed by either CNN or graph convolutional neural networks (GCNN) architectures. Protein complexes are typically represented using strategies based on topological information (87), solvent accessible surface (SAS) (88), voxel-based molecular surface representation (89), and various molecular descriptors (90).

Another of the traditional drug repurposing methods are the gene expression based algorithms (83). The changes in the expression levels of defensive genes in disease states can be used as phenotypic descriptors or quantifiers of the transcriptomic effects of the explored drugs. Besides, methods based on integrated docking simulation algorithms have made it possible to optimize drug repurposing systems (91).

Different computational tools have been developed for drug and vaccine discovery. Zhavoronkov et al. (92) developed a generative chemistry pipeline based on the knowledge of protein, molecule structures, and homology models strategies to identify new drugs related to SARS-CoV-2. Tang et al. (93) have built processes based on deep learning (DL) algorithms to design new antivirus drugs of a chemical or peptide nature based on the information available in the literature and different chemical rules.

Molecular simulations using docking techniques allowed the development of virtual screening methodologies and iterative searches to discover new drugs of interest. The discovery of new chemical compounds with desirable activities is possible by combining the structural information with strategies of deep generative models (94, 95).

Predictive models using the linear protein sequences and the chemical compounds represented as SMILES have been proposed to predict affinity between proteins and chemical compounds (96). Different numerical representations strategies have been implemented to encode the protein sequences, such as binarization coding, physicochemical properties, and Fourier transforms to represent protein sequences in spaces of signals (97). Alternatively, methods based on natural language processing (protein language models) have been developed (98). In the case of SMILES, different autoencoders and transformers strategies have been created, including variational autoencoders and graph junction trees (99).

Performance between methods based on linear sequences information and those that only incorporate structural details are similar. However, the processes that use representations based on NLP seem to present a higher performance because the autoencoders manage to learn the structural relationships that guide the function (100). Nevertheless, the learning strategies and the abilities to extract complex patterns from the information used for the development of predictive models are properties of DL methods that, to date, have not been fully understood due to their functioning as black boxes. The incorporation of techniques based on explainable AI, is under development to understand the underlying functions and mechanics of the ML algorithms (101).

Concerning the strategies to prevent the adverse effect provoked by the vaccination programs, ML analyzes revealed distinct arterial pulse variability according to side effects of mRNA vaccine. This can facilitate a time-saving and easy-to-use method for detecting changes in the vascular properties associated with cardiovascular side effects following vaccination (102).

The application of explainable ML techniques has allowed to detect relevant variables to perform predictive models with hight performances. Abbaspour et al. (103) applied SHAP strategies combined with XGB model to identify important predictors (e.g., demographics, any history of allergy, any prior COVID diagnosis or positive test, vaccine manufacturer, and time-of-day-of-vaccination) associated to COVID-19 vaccine-related side effects.

Analyzes of the Vaccine Adverse Event Reporting System datasets with ML and a statistical approaches identified and classified pre-existing factors as having an impact on post-vaccination morbidity and reactogenicity (104). Nevertheless, this information is limited because the main databases do not have a larger record size and do not cover all types of vaccines, provoking problems in the generalization of the identified behaivors.

### 2.3. COVID-19 epidemiology

The design and implementation of ML models used for predicting epidemiological variables was a significant challenge. The need of high volumes of data to generalize the behavior of the predictive models (105), made necessary to develop methods for optimizing the representation of the inputs by autoencoders or embedding (106). The developed models were generated to promote the implementation of computer systems for the simulation of scenarios (107) and to facilitate the elaboration of government public policies focused on preventing the increase in the number of contagious or the outbreak of new waves (108).

Depending on the input type, the construction of predictive models can be based on forecasting methods using strategies such as ARIMA (109, 110) or LSTM architectures (111). Other strategies were based on logistic regression methods (112), nonlinear regressions (113), autoregressive models (114), and Gaussian Process Regression (115). The inputs used to develop the predictive models contemplate information based on time series and consider contagion spread records, NPI, scenarios, and different types of crucial information related to epidemiological variables. Mathematical methods based on linear algebra and kernel applications were used to combine the different kinds of data in hybrid systems elaborated with RNN and CNN architectures (116).

### 2.4. COVID-19 public health

One essential use of ML strategies was combining mathematical models to develop hybrid knowledge systems to support decisions in public health. These systems can be classified mainly into monitoring applications and simulation systems (116). Concerning monitoring tools, predictive models allow the generation of early alerts of behaviors during a pandemic. These alerts were usually related to predicting waves and new contagion outbreaks. More limited strategies but with significant impact were the methodologies to forecast the level of ICU occupancy in hospitals and health systems and their correlation with increases in contagion rates and mutational variants since it allowed early warning of the occupancy level and facilitated decision-making to prevent a whole occupancy level (117).

The simulation of scenarios by ML allowed the evaluation of public policy effect on populations of interest (118). Despite the versatility of ML, dynamic changes in the knowledge embedded in the system—NPI modifications, the application of vaccine programs, emergence of SARS-CoV-2 variants, etc- makes necessary a constant adaptation of ML based models. Incorporation of reinforced learning might help to facilitate this process.

### 2.5. Application to long COVID

With the emergence of LC, ML methods have been employed for the development of predictive tools, the construction of statistical systems for relating patient phenotypes, and the elaboration of rules and complex patterns to understand the interactions between systems and types of sequelae. The application of unsupervised learning algorithms like *k*-means and kernel representations strategies enabled to correlate symptoms and different classifications of LC (119).

Based on data from the N3C electronic health record repository, Pfaff et al. (119) have developed an ML model to classify the likelihood of LC diagnosis. Using XGBoost machine learning algorithm this study identified a series of features, including the healthcare utilization rate, patient age, dyspnea or respiratory symptoms, other pre-existing risk factors (diabetes, kidney disease, congestive heart failure, or pulmonary disease), and treatment medication information to predict LC.

Binka et al. (120) proposed a classification model based on elastic net penalized logistic regression algorithms for classifying patients as positive or negative for LC. The model proposed by Binka et al. (120) employed as descriptors demographic characteristics, pre-existing conditions, COVID-19 related data, and all symptoms/conditions recorded >28–183 days after the COVID-19 symptom onset/reported.

Fritsche et al. (121) described associations from the previous and acute medical phenomena of COVID-19 as predisposing diagnoses for LC employing statistical and relation features models.

Performed phenomenon-wide association studies (PheWa) and Phenotype Risk Scores (PheRS) have uncovered a plethora of diagnoses associated with LC. These studies associated seven phenotypes with the pre-COVID-19 period (e.g., irritable bowel syndrome, concussion, nausea/vomiting, and shortness of breath) and 69 acute-COVID-19 phenotypes (predominantly respiratory and circulatory phenotypes) significantly associated with LC. Using PheRS, a quarter of the COVID-19 positive cohort was identified with a 3.5-fold increased risk of LC compared to the bottom 50% of their distributions (121).

Sengupta et al. (122) proposed an interpretable DL approach based on Gradient-weighted Class Activation Mapping using N3C and RECOVER data to predict risk factors contributing to the development of LC. This model used a temporally ordered list of diagnostic codes six weeks post-COVID-19 infection for each patient, with an accuracy of 70.48%. Gupta et al. (123) proposed a stacking ensemble learning technique based on deep neural networks for early predicting cardiovascular disease risk in recovered SARS-CoV-2 patients with LC symptoms, achieving an accuracy of 93.23%.

The here reviewed studies highlight the versatility of ML methods to study LC, facilitating not only the implementation of predictive diagnostic tools but also encouraging the integration of clinical data with, social, demographic and other information, for the development of robust systems. Despite the versatility of ML techniques, there are still enormous challenges for their application in LC analysis, in particular the collection of meaningful data sets for the development of predictive systems.

## 3. Discussion

Mathematical models have helped to understand the dynamics of the spread of SARS-CoV-2 and helped to predict different scenarios during the COVID-19 pandemic, becoming one of the most relevant tools for developing public health policies. Correlating sanitary measures with virus variants and the effects on the reproduction rate enabled the assessment of government policies that will help to face new outbreaks of SARS-CoV-2 or future pandemics. The development of reliable mathematical models, statistical techniques for test correction, and methods of analysis of heterogeneous populations, together with the value of testing strategies and traceability of close contacts, has been remarkable achievements. Combining these systems with ML and AI methods increased the predictive power of the models and facilitated the simulation of scenarios.

Developing predictive systems for COVID-19 was one of the significant challenges assumed by thousands of scientists during the pandemic. The main achievements were developing models for clinical diagnostic systems, ML for drug and vaccine discovery, and forecasting models for epidemiological variables to support public health policies and monitoring systems. In turn, the development of predictive systems coupled with techniques such as protein language models and molecular techniques facilitated the study of variants at the genomic level. Such models helped to understand how mutations affected critical viral proteins, helping drug and vaccine designs.

The ongoing pandemic has introduced a complete set of challenges, and currently, a novel multisystem disease defined by the persistence or appearance of new symptoms after SARS-CoV-2 infection has emerged. This complex entity-denominated LC has yet to be fully elucidated, mainly because it is characterized by a wide range of clinical manifestations, methodological limitations, and heterogeneous definitions that make clinical and computational analysis difficult. Despite rapidly emerging studies and growing evidence, current data needs to be improved. A primary task is to establish an approach to identify natural language data associated with potential LC patients. This task will likely require well-designed prospective studies, unified definitions of LC, an accurate distinction of SARS-CoV-2-related symptoms, and adequate follow-up times that include current patients, underrepresented groups, children, and minority populations. It is granted that ML strategies will play a critical role in the understanding of LC and other upcoming challenges of the ongoing SARS-CoV-2 pandemic.

## Author contributions

LS, JG-P, and DM-O: conceptualization. DM-O, DA-S, and JA: methodology. DM-O and MN: validation. LS, JG-P, DM-O, JA, and DA-S: investigation. LS, DM-O, JG-P, and MN: writing, review, and editing. MN and RU-P: supervision, funding resources, and project administration. All authors contributed to the article and approved the submitted version.
